# Further study of two Chinese cave spiders (Araneae, Mysmenidae), with description of a new genus

**DOI:** 10.3897/zookeys.870.35971

**Published:** 2019-08-07

**Authors:** Chengcheng Feng, Jeremy A. Miller, Yucheng Lin, Yunfei Shu

**Affiliations:** 1 Key Laboratory of Bio-Resources and Eco-Environment of Ministry of Education, College of Life Sciences, Sichuan University, Chengdu 610065, Sichuan, China Sichuan University Sichuan China; 2 Department of Biodiversity Discovery, Naturalis Biodiversity Center, Postbus 9517 2300 RA Leiden, The Netherlands Naturalis Biodiversity Center Leiden Netherlands

**Keywords:** China, Gaoligong Mountains, *
Maymena
*, new genus, phylogeny, symphytognathoids, troglobite

## Abstract

The current paper expands knowledge of two Chinese cave spider species originally described in the genus *Maymena* Gertsch, 1960: *M. paquini* Miller, Griswold & Yin, 2009 and *M. kehen* Miller, Griswold & Yin, 2009. With the exception of these two species, the genus *Maymena* is endemic to the western hemisphere, and new evidence presented here supports the creation of a new genus for the Chinese species, which we name *Yamaneta***gen. nov.** The male of *Y. kehen* is described for the first time. Detailed illustrations of the habitus, male palps and epigyne are provided for these two species, as well as descriptions of their webs. DNA sequences are provided for both *Yamaneta* species. We build on a previously published phylogenetic analysis of Mysmenidae to assess the phylogenetic position of *Yamaneta* and its relationship to true *Maymena*.

## Introduction

The genus *Maymena* Gertsch, 1960 was established in the context of a taxonomic paper describing several American spiders of the family Symphytognathidae Hickman, 1931. At that time, the concept of Symphytognathidae was broader than it is today; the taxa described therein are currently distributed among four families (Symphytognathidae, Mysmenidae Petrunkevitch, 1928, Anapidae Simon, 1895 and Theridiosomatidae Simon, 1881). The world’s Symphytognathidae were reviewed and redefined by Forster and Platnick (1977), and several symphytognathid genera were transferred to other families, including *Maymena* to the Mysmenidae. Gertsch’s (1960) original description included observations of several characteristics of *Maymena* but did not provide diagnostic characters for separating the genus from its close relatives. It was not until the recent publication of Lopardo and Hormiga (2015) that a rigorous and convincing diagnosis of *Maymena* was finally published.

Miller et al. (2009) described several symphytognathoid spiders from the Gaoligong Mountains, Southwest China. Two species from caves were placed in the genus *Maymena*: *M. paquini* Miller, Griswold & Yin, 2009 and *M. kehen* Miller, Griswold & Yin, 2009, the latter species being known only from females. The genus *Maymena* currently contains 13 described species (World Spider Catalog 2019). Except for the two Chinese species, all are known from the western hemisphere, from the USA south through Mexico, Central America, the Caribbean, and Peru. In addition, two undescribed taxa in Lopardo et al. (2011) considered to belong to *Maymena* (Lopardo and Hormiga 2015) were from Argentina. Most *Maymena* species are clearly associated with caves, although a few species are occasionally or typically found in surface habitats (Gertsch 1960, 1971, Brignoli 1974, Baert 1990, Eberhard et al. 1993).

In August 2008, students and professors of Sichuan University carried out a collecting survey in the Gaoligong Mountains. Both males and females of Miller et al.’s Chinese *Maymena* species were collected from their type localities and their web structures were discovered and photographed. In addition to new detailed morphological data and the description of the previously unknown male of *M. kehen*, multiple individuals of both species were sequenced for five loci. To test the relationships of Chinese *Maymena* to western *Maymena* and other Mysmenidae, we added this DNA sequence data to the molecular phylogenetic dataset of Lopardo et al. (2011).

## Material and methods

Specimens were acquired by hand from the dark zone of caves and preserved in 95% ethanol. They were examined using a Leica M205 C stereomicroscope. Further details were studied under an Olympus BX43 compound microscope. Male palps and epigynes were examined and photographed after dissection. Epigynes were treated in lactic acid before being embedded in Arabic gum to take the photos of the vulva. To reveal the course of the spermatic ducts, male palps were also clarified using lactic acid and subsequently mounted in Hoyer’s Solution. The left palp was photographed and described. Photos were taken with a Canon EOS 60D wide zoom digital camera (8.5 megapixels) mounted on an Olympus BX 43 compound microscope. The images were montaged using Helicon Focus 3.10 (Khmelik et al. 2006) image stacking software. All measurements are in millimeters. Leg measurements are given as follows: total length (femur, patella, tibia, metatarsus and tarsus).

Tissue samples were taken from eight individual specimens of Chinese *Maymena* representing both known species. Whole genomic DNA was extracted from tissue samples with TIANamp Micro DNA Kit (TIANGEN) following the manufacturer’s protocol for animal tissues. Five gene fragments were amplified in 25μL reactions: mitochondrial large-subunit ribosomal RNA (16S), nuclear small-subunit ribosomal RNA (18S), nuclear large-subunit ribosomal RNA (28S), cytochrome *c* oxidase subunit I (COI), and histone H3 (H3). Primer pairs and PCR protocols are given in Table 1. Raw sequences were edited and assembled using BioEdit v.7.2.5 (Hall 1999). New sequences generated for this study were deposited in GenBank; accession numbers are reported in Table 2. All molecular vouchers and examined materials are deposited in the Natural History Museum of Sichuan University in Chengdu (**NHMSU**), China.

**Table 1.** The loci, primer pairs, and PCR protocols used in this study.

**Locus**	**Annealing temperature/time**	**Direction**	**Primer**	**Sequence 5’→3**’	**Reference**
16S	48.5°/30s	F	LR-J-12864	CTCCGGTTTGAACTCAGATCA	Hormiga et al. 2003
		R	LR-J-13360	GTAAGGCCTGCTCAATGA	This study
	45°/30s	F	LR-J-12964	AACTCAGATCATGTAATAATT	This study
		R	LR-J-13360	GTAAGGCCTGCTCAATGA	This study
18S	54.9°/30s	F	18S-1F	TACCTGGTTGATCCTGCCAGTAG	Giribet et al. 1996
		R	SSU rRNA reverse	GTGGTGCCCTTCCGTCAATT	Balczun et al. 2005
28S	53.1°/30s	F	28Sa	GACCCGTCTTGAAACACGGA	Rix et al. 2008
		R	LSUR	GCTACTACCACCAAGATCTGCA	Rix et al. 2008
COI	46°/30s	F	LCO1490	GGTCAACAAATCATAAAGATATTGG	Folmer et al. 1994
		R	HCO2198	TAAACTTCAGGGTGACCAAAAAATCA	Folmer et al. 1994
	45°/30s	F	LCO1490	GGTCAACAAATCATAAAGATATTGG	Folmer et al. 1994
		R	C1-N-2191 (Nancy)	CCCGGTAAAATTAAAATATAAACTTC	Simon et al. 1994
H3	46°/30s	F	H3aF	ATGGCTCGTACCAAGCAGACVGC	Colgan et al. 1998
		R	H3aR	ATATCCTTRGGCATRATRGTGAC	Colgan et al. 1998
	49.4°/30s	F	H3nF	ATGGCTCGTACCAAGCAGAC	Colgan et al. 1998
		R	H3nR	ATRTCCTTGGGCATGATTGTTAC	Colgan et al. 1998

**Table 2.** GenBank accession numbers for new DNA sequence data provided here.

**Species**	**Identifier**	**Sex/Stage**	**16S**	**18S**	**28S**	**COI**	**H3**
*Yamaneta kehen*	GlgMY14	Male	MK908789	MK908805	MK908797	MK895530	MK895538
	GlgMY14	Female	MK908790	MK908806	MK908798	MK895531	MK895539
	GlgMY14	Juvenile	MK908791	MK908807	MK908799	MK895532	MK895540
	GlgMY15	Male	MK908792	MK908808	MK908800	MK895533	MK895541
	GlgMY15	Female	MK908793	MK908809	MK908801	MK895534	MK895542
*Yamaneta paquini*	GlgMY16	Male	MK908794	MK908810	MK908802	MK895535	MK895543
	GlgMY16	Female	MK908795	MK908811	MK908803	MK895536	MK895544
	GlgMY16	Juvenile	MK908796	MK908812	MK908804	MK895537	MK895545

The most recent molecular phylogeny of Mysmenidae was Lopardo et al. (2011). Lopardo supplied alignments of the six genes used in their analysis (the five above plus the mitochondrial small-subunit ribosomal RNA 12S). Taxonomic determinations were updated according to notes in Lopardo and Hormiga (2015). The COI sequence “Mysmena-MYSM-018-MAD” (GU456888) was omitted because it was flagged on GenBank as removed at the submitter’s request because of possible contamination. We used the MAFFT version 7 online service (https://mafft.cbrc.jp/alignment/server/add_sequences.html) with the following settings to add the Chinese *Maymena* sequences to the existing alignments of the five shared loci (Strategy: Auto, scoring matrix for nucleotide sequences: 200PAM/k=2, Gap opening penalty: 1.53, offset value: 0.0; Katoh et al. 2017). Alignments of all six loci were concatenated in Geneious version 8.1.8 (https://www.geneious.com). The final alignment consisted of 6038 positions (Suppl. material 1). Uncorrected pairwise distances between terminals in the expanded alignment were calculated using MEGA X (Kumar et al. 2018) and are included as Suppl. material 2. We did not build on the morphological data matrix of Lopardo et al. (2011) or the expansion of this dataset in Lopardo and Hormiga (2015), and these data were not used in our analysis. This is because DNA sequence datasets are relatively simple to expand with additional data; it would be difficult for us to do the same for the large and complex morphological dataset without risking the introduction of errors and artifacts.

The most parsimonious tree was found using 1000 replicates of random taxon addition and TBR (Tree-Bisection-Reconnection) branch swapping using MEGA X (Kumar et al. 2018). To assess support, bootstrap values were calculated using MEGA X (Kumar et al. 2018) with 1000 bootstrap replicates, each consisting of 1000 replicates of random taxon addition and TBR branch swapping. Departing from the approach of Lopardo et al. (2011), gaps/missing data were treated as ambiguities, not as a 5^th^ character state.

The Bayesian phylogenetic inference was performed using MrBayes version 3.2.6 (Ronquist and Huelsenbeck 2003) through the Cipres Science Gateway (Miller et al. 2010). Partitions and models followed Lopardo et al. (2011; table 4). A total of 10 partitions were defined (three independently modeled regions each for the nuclear ribosomal genes 18S and 28S, single model for each of the remaining four loci). The Bayesian search consisted of 50,000,000 generations using four chains, with the chain sampled every 1000 generations (see Suppl. material 1). Tracer version 1.7.1 (Rambaut et al. 2018) was used to establish the appropriate level of burn-in, which was set at 5000.

Abbreviations appearing in text and figures are as follows:

**ALE** anterior lateral eyes

**AME** anterior median eyes

**BC** base of cymbium

**BH** basal haematodocha

**CA** cymbial apophysis

**CD** copulatory ducts

**CS** clasping spine on leg I

**Cy** cymbium

**CyC** cymbial conductor

**CyFs** setae on cymbial fold

**E** embolus

**FD** fertilization ducts

**PC** paracymbium

**PLE** posterior lateral eyes

**PME** posterior median eyes

**S** spermathecae

**SD** spermatic duct

**Sp** scape

**T** tegulum

**Ti** tibia

**TiS** setae on palpal tibia

**TS** tibial spine on leg I

**TTr** trichobothria on tibia

Institutional acronyms:

**NHMSU** Natural History Museum of Sichuan University, Chengdu, China

## Results

**Figure 1.**
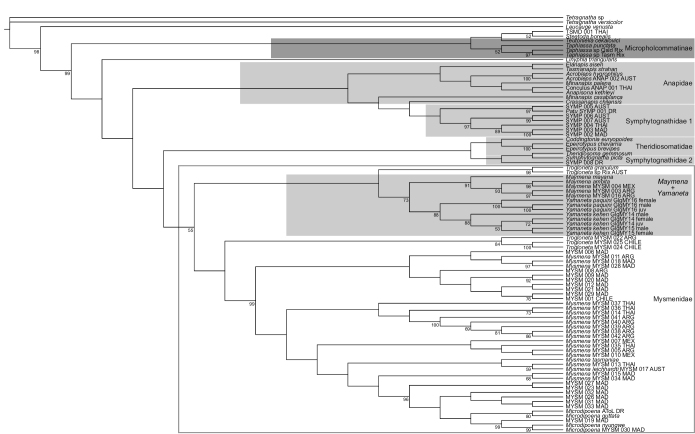
Single most parsimonious tree (15922 steps) resulting from the analysis of Lopardo et al.’s (2011) “Molecular A” alignment plus 8 new sequences from *Yamaneta* gen. nov. specimens. Numbers at nodes indicate bootstrap support ≥ 50%. Family Mysmenidae indicated with a gray box; clade *Maymena+Yamaneta*, and also families Anapidae, Symphytognathidae, and Theridiosomatidae (except for TSMD 001 THAI) indicated with light gray shading, anapid subfamily Micropholcommatinae indicated with dark gray shading. Note non-monophyly of Anapidae, Symphytognathidae, Theridiosomatidae, and Micropholcommatinae.

Parsimony analysis of the expanded sequence alignment recovered a single most parsimonious tree (Fig. 1). This tree features a monophyletic, but weakly supported, Mysmenidae. Western hemisphere and Chinese *Maymena* are reciprocally monophyletic, with moderate bootstrap support. The two Chinese species are coherent. Few clades, especially along the backbone of the phylogeny, have high bootstrap support, and relationships among outgroup taxa are complicated. The low support values seem in part to be attributable to a number of unstable taxa. *Maymena* (western and Chinese) and *Trogloneta* Simon, 1922 together form a paraphyletic complex, with one branch of *Trogloneta* sister to *Maymena* (western and Chinese) and the other *Trogloneta* branch sister to the remaining Mysmenidae.

**Figure 2.**
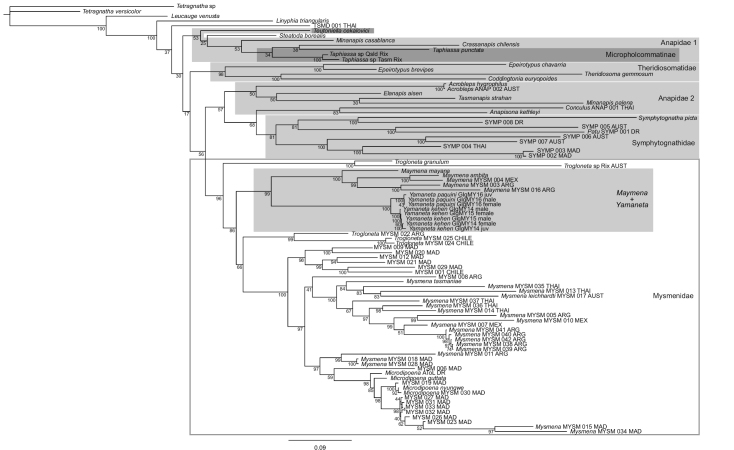
Topology from Bayesian mixed model analysis based on Lopardo et al.’s (2011) “Molecular A” alignment plus 8 new sequences from *Yamaneta* gen. nov. specimens. Numbers at nodes indicate percent posterior probabilities; other conventions as in Fig. 1. Note diphyly of Anapidae, nesting of most Micropholcommatinae within “Anapidae 1”, and placement of *Teutoniella cekalovici* outside other micropholcommatines.

After 50,000,000 generations of Bayesian analysis, the average deviation of split frequencies fell below 0.05. The combined effective sample sizes of the two MCMC chains were 7425.9 and 7654.5 (12,520.9 combined), comfortably above the recommended minimum of 200 (Lanfear et al. 2016). The Bayesian topology (Fig. 2) features a monophyletic Mysmenidae, which in contrast to the parsimony analysis enjoys high support from posterior probability. As in the parsimony analysis, western and Chinese *Maymena* are reciprocally monophyletic with high support, and the two Chinese species are coherent. However, branch lengths suggest a long separation between the Chinese and western lineages. The Bayesian analysis also indicates complicated relationships among the outgroup taxa. *Maymena* (western and Chinese) and *Trogloneta* together form a paraphyletic complex similar to that found in the parsimony analysis; one branch of *Trogloneta* is sister to all other Mysmenidae, and the other branch of *Trogloneta* is sister to the remaining Mysmenidae, except for *Maymena* (western and Chinese).

## Discussion

Monophyly of and relationships between the so-called symphytognathoid families (including Mysmenidae, Anapidae, Theridiosomatidae and Symphytognathidae) are complicated and inconsistent across various analyses. Early attempts based on morphological data (e.g., Griswold et al. 1998) proposed a “symphytognathoid” clade composed of monophyletic families, but Schütt (2003) warned that some of this could be based on a syndrome of parallel reductions and simplifications related to the evolution of small size. DNA sequences initially seemed promising as a source of phylogenetic data independent of morphology, where homology assessment could be confused with parallel evolution. The analysis of Rix et al. (2008) tested the relationships of Anapidae and Micropholcommatidae (currently considered part of Anapidae; Schütt 2003, Lopardo et al. 2011, World Spider Catalog 2019), with representatives of several other relevant families, based on DNA sequence data from two nuclear ribosomal loci. Results concerning the primary focal group of the study, the Micropholcommatidae, were fairly robust and consistent. However, results regarding the Anapidae and key outgroup taxa were generally of poor resolution and inconsistent across tree-building methods. This despite being built upon what was, for the time at least, a rigorous and sophisticated analytical approach. The results of the analyses reported in Lopardo et al. (2011) were similarly sensitive to changes in analytical parameters. This analysis was based on an expanded set of loci compared to Rix et al. (2008), plus a set of morphological characters, and many permutations of data partitions and phylogenetic optimization methods were employed. The monophyly of Mysmenidae was relatively robust to permutations of the analysis, but the inclusion of morphological data had a tendency to support the monophyly of outgroup families, which sometimes collapsed in analyses based on molecular sequences alone. A series of studies by Dimitrov et al. (2012, 2016) used roughly the same set of loci with a progressively expanded sample of taxa to explore deep questions of spider relationships. These consistently recovered two clades of anapids and never found support for the monophyly of symphytognathoid families. The phylogenetic analysis of Wheeler et al. (2017) further expanded the taxon sample, but not the selection of loci. Anapidae (including a monophyletic Micropholcommatinae), Symphytognathidae, and Mysmenidae were each monophyletic and moderately well supported; Theridiosomatidae was monophyletic only after the pruning of one problematic taxon, and still presented with low support. The four symphytognathoid families together were found to be closely related but not monophyletic.

The parsimony and Bayesian phylogenies presented here disagree about outgroup relationships in several important ways, including the monophyly of Anapidae, its relationship to Micropholcommatinae, and the sister clade to Mysmenidae. Such results are not surprising, because previous studies relying on the same limited set of reliable loci have seen similar results for nearly a decade, and also because taxon sampling outside Mysmenidae in this study and its predecessors (Lopardo et al. 2011, Lopardo and Hormiga 2015) is very limited.

Recent phylogenomic approaches have finally expanded the volume of DNA sequence data used to investigate spider phylogeny (Bond et al. 2014, Fernández et al. 2014, 2018, Garrison et al. 2016), but only Fernández et al. (2018) has achieved the taxon sampling necessary to address some of the longstanding symphytognathoid questions. Their study, based on ca 2500 genes, found monophyletic Theridiosomatidae (4 terminals), Mysmenidae (3 terminals) and Anapidae (2 terminals; Symphytognathidae was not represented); none of the symphytognathoid families present were found to be sister taxa. We look forward to further studies with greatly expanded DNA sequence coverage and the taxon sample necessary to address longstanding symphytognathoid questions.

**Figure 3.**
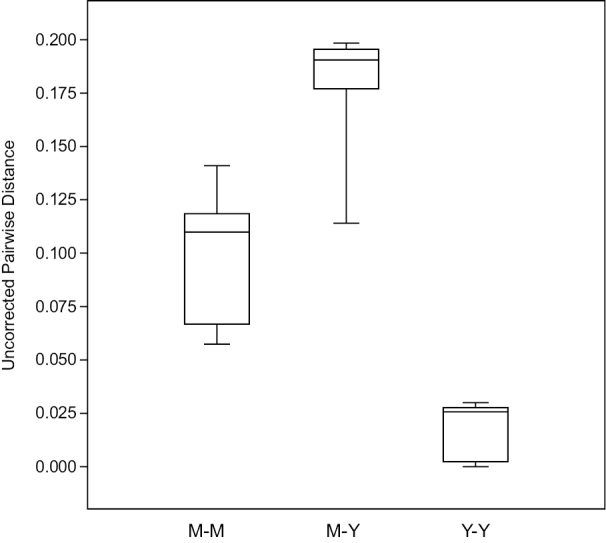
Box plot of uncorrected pairwise distances between terminals representing *Maymena* (M-M), *Yamaneta* gen. nov. (Y-Y), and between *Maymena* and *Yamaneta* (M-Y). See Suppl. material 2 for complete distance matrix.

Lopardo and Hormiga (2015) noted that the placement of the several mysmenid species and genera described by Miller et al. (2009) had yet to be tested phylogenetically; here we have begun to rectify this. Both parsimony and Bayesian analyses found the Chinese *Maymena* species formed a clade sister to the remaining *Maymena*. This suggests that placing the Chinese species in *Maymena* was defensible. However, multiple lines of available evidence seem sufficient to justify the creation of a new genus to accommodate the Chinese species. The two Chinese *Maymena* are the only members of the genus known from beyond the western hemisphere. Although the Chinese *Maymena* resemble and share several characters with those from the west (e.g., aspects of the genital morphology, trichobothria on the male palpal tibia, troglophily, web architecture), they also express distinguishing features (e.g., elongate male palpal tibia and patella, relatively long and setose epigynal scape). Bayesian branch lengths (Fig. 2) and uncorrected pairwise distances based on our alignment (Fig. 3) both indicate a degree of distinctness between the Chinese and western *Maymena*.

## Taxonomy

### Mysmenidae Petrunkevitch, 1928

#### Genus Yamaneta

Taxon classificationAnimaliaAraneaeMysmenidae

Miller & Lin gen. nov.

1DE18130FDDA50BFBC427EE47C0D9D0A

http://zoobank.org/5383A9BC-F125-4D4B-8603-BDA44B06163D

##### Type species.

*Maymena paquini* Miller, Griswold & Yin, 2009.

##### Etymology.

Formed from *Yama*, the figure in Chinese mythology who oversees the realm of the dead, and -*neta* (-νήτης), an element in several spider names conventionally taken to mean ‘spinner’ (Cameron 2005). The gender is masculine.

##### Diagnosis.

Distinguished from other mysmenid genera except *Maymena* by the presence of a modified spatulate seta on the PLS (Miller et al. 2009: fig. 57D, F; Lopardo and Hormiga 2015: fig. 11G, H), the proximal position of the male metatarsus I clasping spur (more proximal in *Maymena* than *Yamaneta*; Miller et al. 2009: fig. 53A; Lopardo and Hormiga 2015: fig. 16G), the shape of the apical part of the cymbium, which appears to form a functional conductor (Miller et al. 2009: fig. 55A; Lopardo and Hormiga 2015: fig. 10D, G) that interacts with the embolus. The presence of trichobothria on the male palpal tibia is a rare character in Mysmenidae, occurring in such genera as *Maymena*, *Yamaneta*, *Trogloneta*, *Mysmenopsis* Simon 1898 and *Isela* Griswold, 1985. Distinguished from *Maymena* by the elongate male palpal tibia and patella, long and setose epigynal scape, by the absence of a modified seta with a long row of branches near the major ampullate gland spigot on the anterior lateral spinnerets (Miller et al. 2009: fig. 57B), and by the clustered arrangement of male epiandrous fusules (Miller et al. 2009: fig. 56D; dispersed in *Maymena*: Lopardo and Hormiga 2015: figs 12B, 16A). The type species *Maymena mayana* (Chamberlin & Ivie, 1938) has been described as having a small rounded scape (Gertsch 1960), although this is a glabrous structure (setose in *Yamaneta*), and *M. mayana* is coded as absent for a scape in phylogenetic data matrices (Lopardo et al. 2011, Lopardo and Hormiga 2015: character 60). There are also similarities in the female reproductive path shared between *M. mayana* and *Yamaneta*, such as the fertilization ducts arising from the copulatory ducts rather than the spermathecae (Lopardo and Hormiga 2015: fig. 128B); internal female reproductive structures and spinneret spigot morphology have been documented for only a few *Maymena* species.

##### Description.

**Figure 4.**
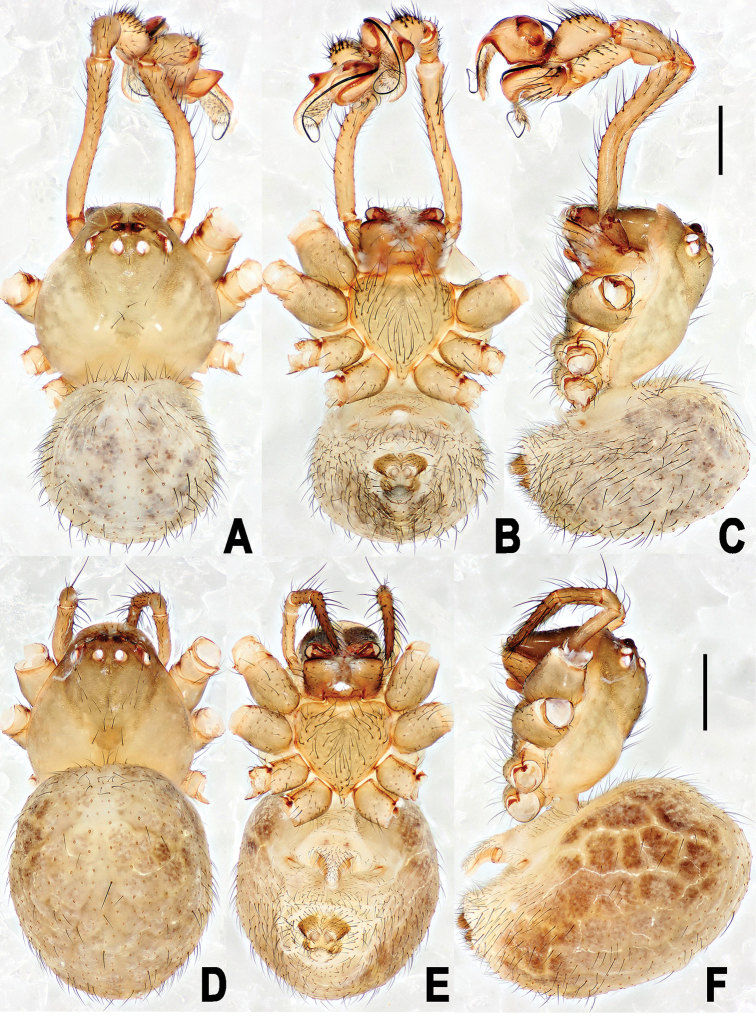
*Yamaneta kehen* (Miller, Griswold & Yin, 2009) comb. nov. from Fugong Co., Lishadi, “a nameless cave” **A–C** Male habitus **D–F** female habitus **A, D** dorsal **B, E** ventral **C, F** lateral. Scale bars: 0.50 mm.

Relatively large mysmenids (>2 mm). Femoral spots on legs I and II in female, leg I only in male. Legs with macrosetae on the femora, tibiae, and metatarsi, especially in the anterior legs. Male clasping spurs arise from distal part of tibia I and basal third of metatarsus I. Leg formula IV-I-II-III. Carapace subovate, ocular area slightly raised. Eight eyes in two rows. AME black and with dark base, others reflective. ALE and PLE contiguous. ARE procurved, PRE straight (Fig. 4). Clypeus moderately high, inclined from anterior lip to eye region. Cervical groove and thoracic fovea indistinct. Thoracic region flat, smooth, nearly hairless except for the eye region and midline. Chelicerae strong, deeper color than carapace. Endites nearly rectangular. Labium rectangular, fused to sternum. Sternum heart-shaped, flat, hirsute, posterior corner sharp (Figs 4B, E, 7B, E). Abdomen globular dorsally, ovate laterally, mottled light to medium gray or tan, sparsely covered with black setae. Spinnerets distinctly sclerotized, the anteriors larger than the posteriors; colulus small, with two tiny setae; anal tubercle pale yellow (Figs 4, 7). Male palpal patella and tibia elongate, palpal tibia with at least one trichobothrium. Hook-like apophysis on prolateral face of cymbium (Miller et al. 2009: fig. 55C). Cymbium folded distally, forming functional conductor. Tegular conductor absent. Embolus long and filiform arising from proximal part of palpal bulb. Epigyne with setose scape extending nearly to the tracheal spiracle (Figs 4E, F, 7E, F). Scape with notched lateral margins (Figs 6D, 9D), profile distinctly curved at dorsum (Figs 6B, 9B). Spermathecae globular, copulatory ducts arise from mesal part of spermathecae, loop near base of scape, terminate in paired openings near middle of scape (Figs 6C, D, 9C, D). Fertilization ducts arise from copulatory ducts rather than spermathecae (Figs 6D, 9D). Male epiandrous fusules with clustered arrangement (Miller et al. 2009: fig. 56D). PLS with modified spatulate seta (Miller et al. 2009: fig. 57D, F; Lopardo and Hormiga 2015: fig. 11G, H).

##### Composition.

*Yamaneta kehen* (Miller, Griswold & Yin, 2009) comb. nov., *Yamaneta paquini* (Miller, Griswold & Yin, 2009) comb. nov.

##### Distribution.

Gaoligong Mountains, Yunnan, China.

##### Affinity with *Maymena*.

**Figure 5.**
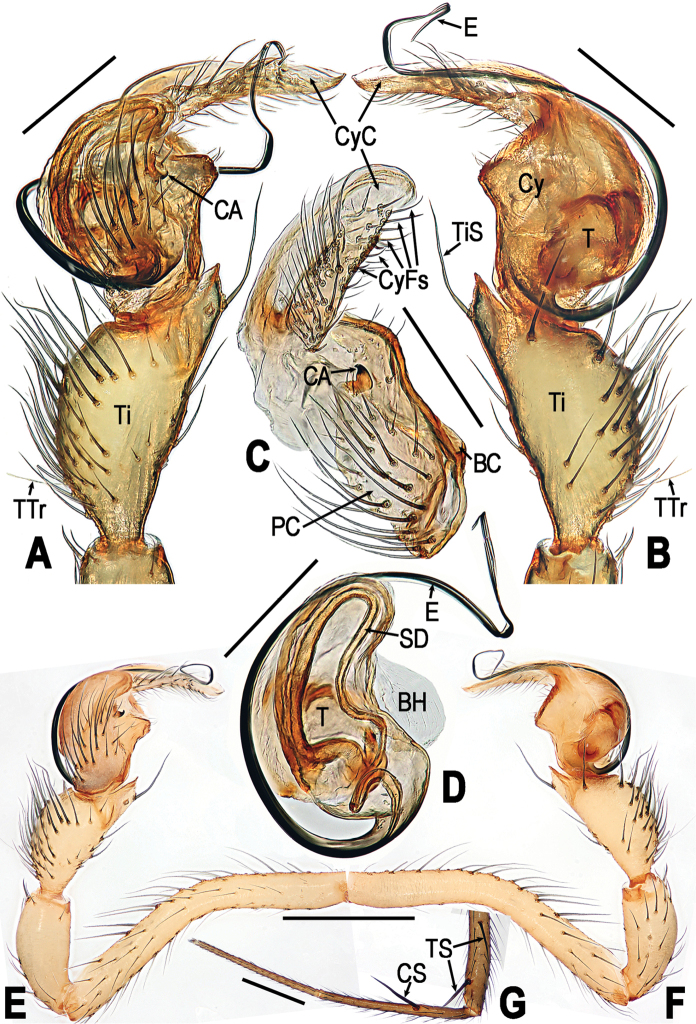
*Yamaneta kehen* (Miller, Griswold & Yin, 2009) comb. nov. from Fugong Co., Lishadi, “a nameless cave”, male **A, B, E, F** Left palp **C** cymbium **D** palpal bulb **G** partial leg I **A, E, G** prolateral **B, F** retrolateral **C** prolateral **D** retrolateral. Abbreviations: **BC** base of cymbium; **BH** basal haematodocha; **CA** cymbial apophysis; **CS** clasping spine on leg I; **Cy** cymbium; **CyC** cymbial conductor; **CyFs** setae on cymbial fold; **E** embolus; **PC** paracymbium; **SD** spermatic duct; **T** tegulum; **Ti** tibia; **TS** tibial spine on leg I; **TTr** trichobothrium on tibia; **TiS** setae on palpal tibia. Scale bars: 0.50 mm.

Lopardo and Hormiga (2015) highlighted several key morphological characteristics of *Maymena* and discussed their status as putative synapomorphies and utility as diagnostic characters. These observations were based on a selection of western species, but many of the characteristics discussed are consistent with *Yamaneta*. The modified spatulate seta on the PLS (Lopardo and Hormiga 2015: fig. 11G, H) is present in *Yamaneta paquini* (Miller et al. 2009: fig. 57D, F [indicated by arrow]). The variable shape of the aciniform gland spigots on both pairs of posterior spinnerets and in both sexes (Lopardo and Hormiga 2015: fig. 11F-H, 13F, G) is visible in *Yamaneta paquini* (Miller et al. 2009: fig. 57C–F). However, the modified seta with a long row of branches near the major ampullate gland spigot on the anterior lateral spinnerets (Lopardo and Hormiga 2015: fig. 11E, 13C, 16B) is not visible in *Yamaneta paquini* (Miller et al. 2009: fig. 57B). The presence of macrosetae on the femora, tibiae, and metatarsi, especially on the anterior legs (Lopardo and Hormiga 2015: figs 140M, 141C), is shared by *Maymena*, *Yamaneta* (Miller et al. 2009: fig. 53A, B, see also text), and the kleptoparasitic clade Mysmenopsinae. A roughly cylindrical palpal tibia (i.e., distal width less than two times proximal width; Lopardo and Hormiga 2015: fig. 10A) is difficult to discern in *Yamaneta*, which have the palpal tibia elongated and modified in shape compared to *Maymena* species (Figs 5, 8). Like *Maymena*, males of *Y. paquini* and *Y. kehen* have a femoral spot on femur I, a clasping spur in a proximal position on male metatarsus I (Miller et al. 2009: fig. 53A; Lopardo and Hormiga 2015: fig. 16G), and lack a tegular conductor. Also consistent across *Maymena* and *Yamaneta* is the presence of macrosetae on the female palpal tarsus (Miller et al. 2009: fig. 53B; Lopardo and Hormiga 2015: figs 13A, 15A). Unlike the *Maymena* species studied by Lopardo and Hormiga (2015: fig. 10H), *Yamaneta* species do not appear to have a deeply grooved embolic rim. As in the *Maymena* species studied by Lopardo and Hormiga (2015), described as having the primary cymbial conductor apically bent over the ventral side (Lopardo and Hormiga 2015: figs 10D, G, 14D), the cymbium of *Yamaneta* species has a complex, almost helical shape, with the embolus and cymbium interacting distally (Figs 5, 8; Miller et al. 2009: figs 54, 55A, B). Unlike *Maymena* (Lopardo and Hormiga 2015: figs 12B, 16A), where the epiandrous fusules are arranged in a dispersed row, those of *Y. paquini* are arranged in a few rough clusters (Miller et al. 2009: fig. 56D). Lopardo and Hormiga (2015: 778) report that the respiratory system of *Maymena* distinguishes it from other mysmenids, but this has not been investigated for *Yamaneta*.

**Figure 6.**
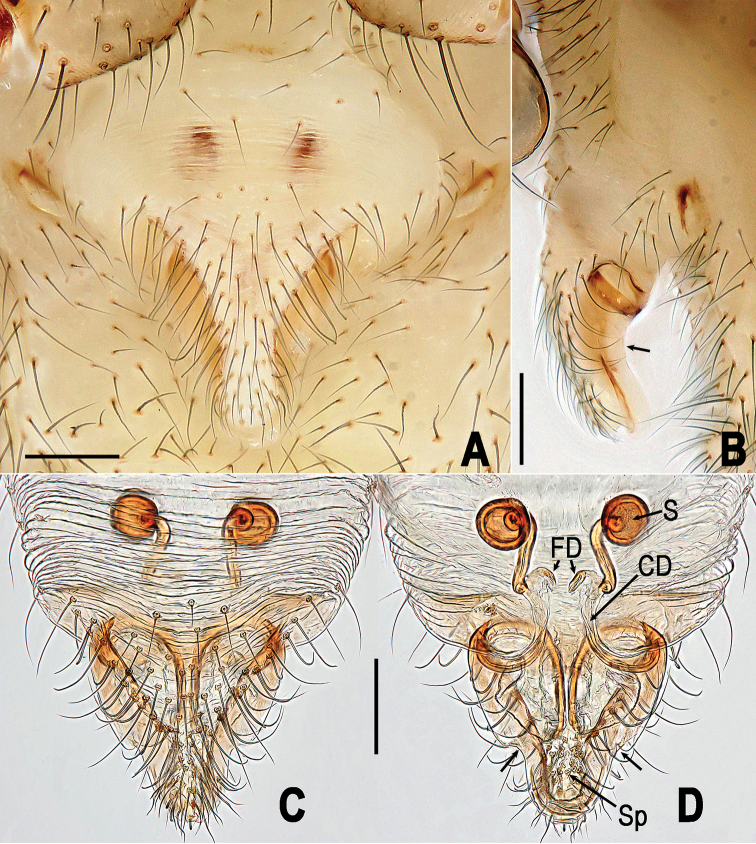
*Yamaneta kehen* (Miller, Griswold & Yin, 2009) comb. nov. from Fugong Co., Lishadi, “a nameless cave”, female genitalia **A, B** Epigyne **C–D** vulva (lactic acid treated) **A, C** ventral **B** lateral **D** dorsal. Unlabeled arrow in **B** indicates curved profile of dorsal surface of scape, in **D** indicates notched lateral margin of scape. Abbreviations: **CD** copulatory ducts; **FD** fertilization ducts; **S** spermathecae; **Sp** scape. Scale bars: 0.10 mm.

**Figure 7.**
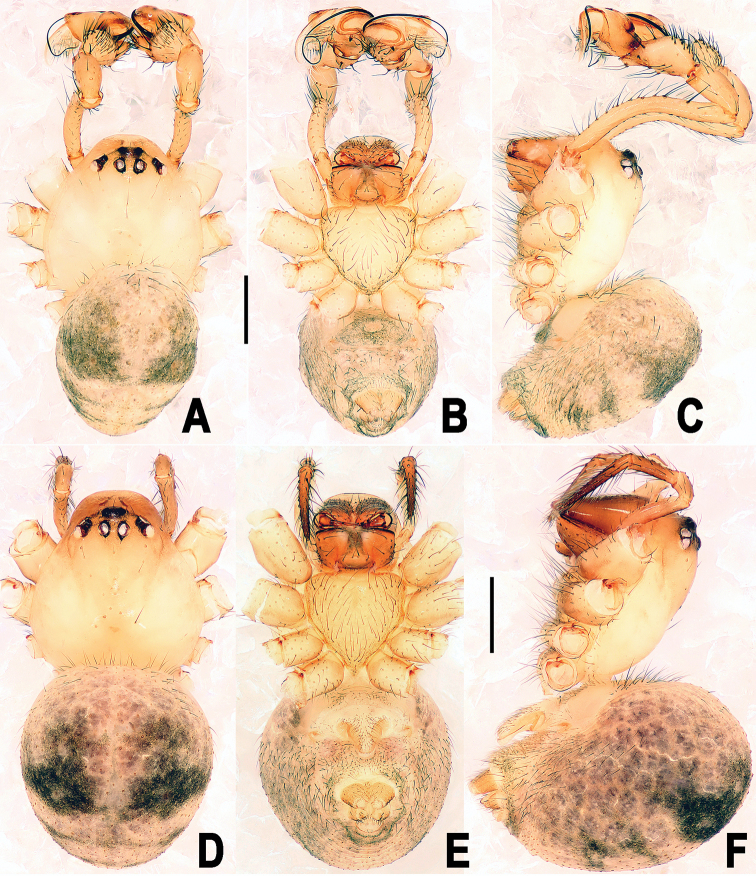
*Yamaneta paquini* (Miller, Griswold & Yin, 2009) comb. nov. from Lushui Co., Daxingdi, Walayaku [cave], male and female **A–C** Male habitus **D–F** female habitus **A, D** dorsal **B, E** ventral **C, F** lateral. Scale bars: 0.50 mm.

**Figure 8.**
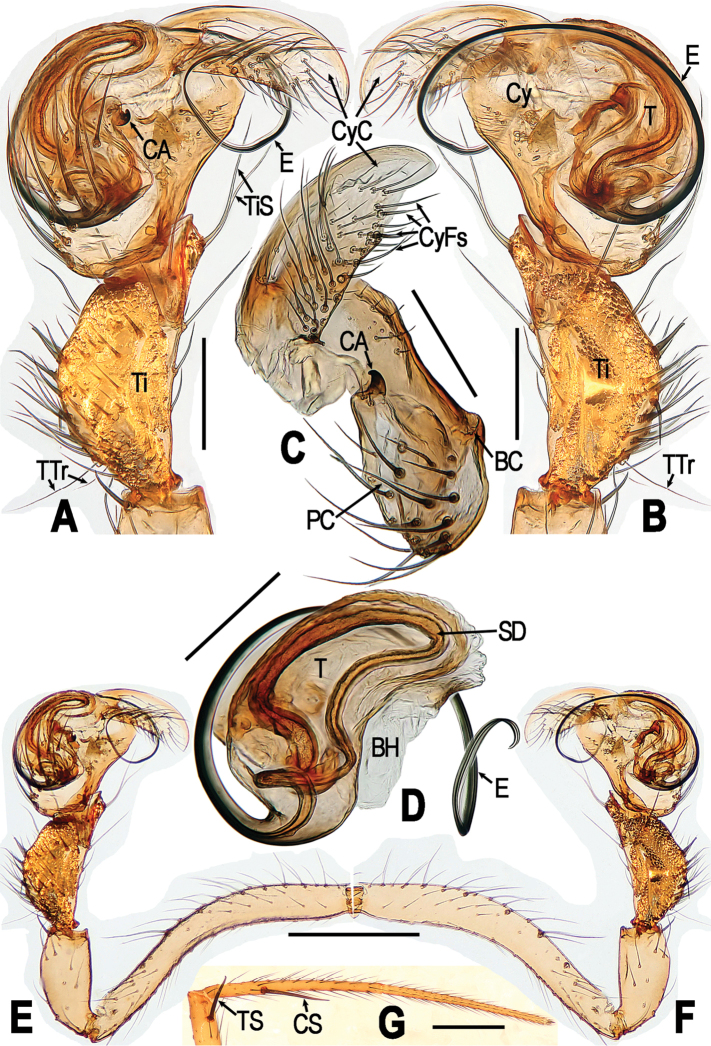
*Yamaneta paquini* (Miller, Griswold & Yin, 2009) comb. nov. from Lushui Co., Daxingdi, Walayaku [cave], male **A, B, E, F** Left palp **C** cymbium **D** palpal bulb **G** partial leg I **A, E, G** prolateral; **B, F** retrolateral; **C** prolateral; **D** retrolateral. Abbreviations: **BC** base of cymbium; **BH** basal haematodocha; **CA** cymbial apophysis; **CS** clasping spine on leh I; **Cy** cymbium; **CyC** cymbial conductor; **CyFs** setae on cymbial fold; **E** embolus; **PC** paracymbium; **SD** spermatic duct; **T** tegulum; **Ti** tibia; **TS** tibial spine on leg I; **TTr** trichobothria on tibia; **TiS** seta on palpal tibia. Scale bars: 0.50 mm.

#### Yamaneta kehen

Taxon classificationAnimaliaAraneaeMysmenidae

(Miller, Griswold & Yin, 2009) comb. nov.

E8616152783D5FAB957D12C72B43096A

Figs 4 , 5 , 6 , 10A

##### Material examined.

CHINA • 2♂♂, 25♀♀ multiple juveniles; Yunnan Province, Nujiang Lisu Autonomous Prefecture, Fugong County, Shiyueliang Town, Lishadi Village, 3.9 km E of Yamu River Fork, “a nameless cave”; 27.12818N, 98.86014E; 1500 m a.s.l.; 18 Aug. 2018; Y.C. Li, Y. Li, Y.F. Shu & Y.C. Lin leg.; NHMSU • 1♂; same data as for preceding; GenBank: MK908789, MK908805, MK908797, MK895530, MK895538; GlgMY14 male • 1♀; same data as for preceding; GenBank: MK908790, MK908806, MK908798, MK895531, MK895539; GlgMY14 female • 1 juvenile; same data as for preceding; GenBank: MK908791, MK908807, MK908799, MK895532, MK895540; GlgMY14 juv. • 1♂; same data as for preceding; GenBank: MK908792, MK908808, MK908800, MK895533, MK895541; GlgMY15 male • 1♀; same data as for preceding; GenBank: MK908793, MK908809, MK908801, MK895534, MK895542; GlgMY15 female.

##### Diagnosis.

**Figure 9.**
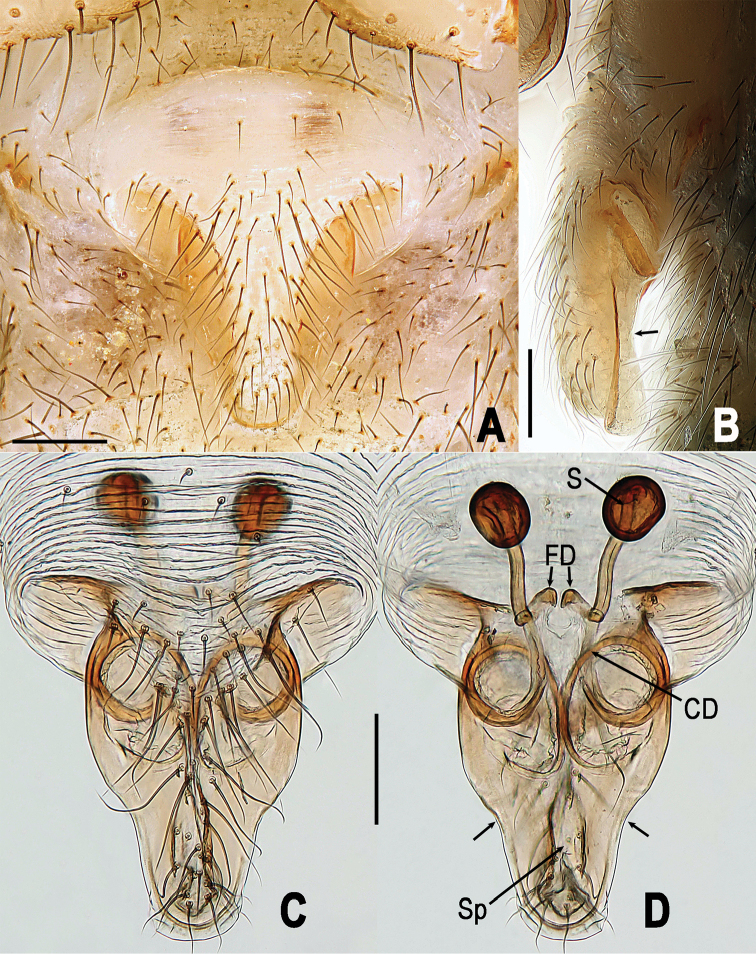
*Yamaneta paquini* (Miller, Griswold & Yin, 2009) comb. nov. from Lushui Co., Daxingdi, Walayaku [cave], female genitalia **A, B** Epigyne **C–D** vulva (lactic acid treated) **A, C** ventral **B** lateral **D** dorsal. Unlabeled arrow in **B** indicates curved profile of dorsal surface of scape, in **D** indicates notched lateral margin of scape. Abbreviations: **CD** copulatory ducts; **FD** fertilization ducts; **S** spermathecae; **Sp** scape. Scale bars: 0.10 mm.

*Yamaneta kehen* can be distinguished from its congener *Y. paquini* by having only a single proximal-dorsal trichobothrium (TTr) and a single long distal-ventral setae (TiS) on the male palpal tibia, but 2 of each in *Y. paquini* (Fig. 5A, B vs. Fig. 8A, B); and by the form of the epigyne in the female, which features a basally wider and shorter scape (Fig. 6C vs. Fig. 9C), a distinctly notched lateral margin of the scape (Fig. 6D vs. Fig. 9D), and a more strongly curved profile of the dorsal surface of the scape (Fig. 6B vs. Fig. 9B). Lateral margins of scape proximal to notches are nearly parallel in *Y. paquini* (Fig. 9C, D), converging in *Y. kehen* (Fig. 6C, D).

**Figure 10.**
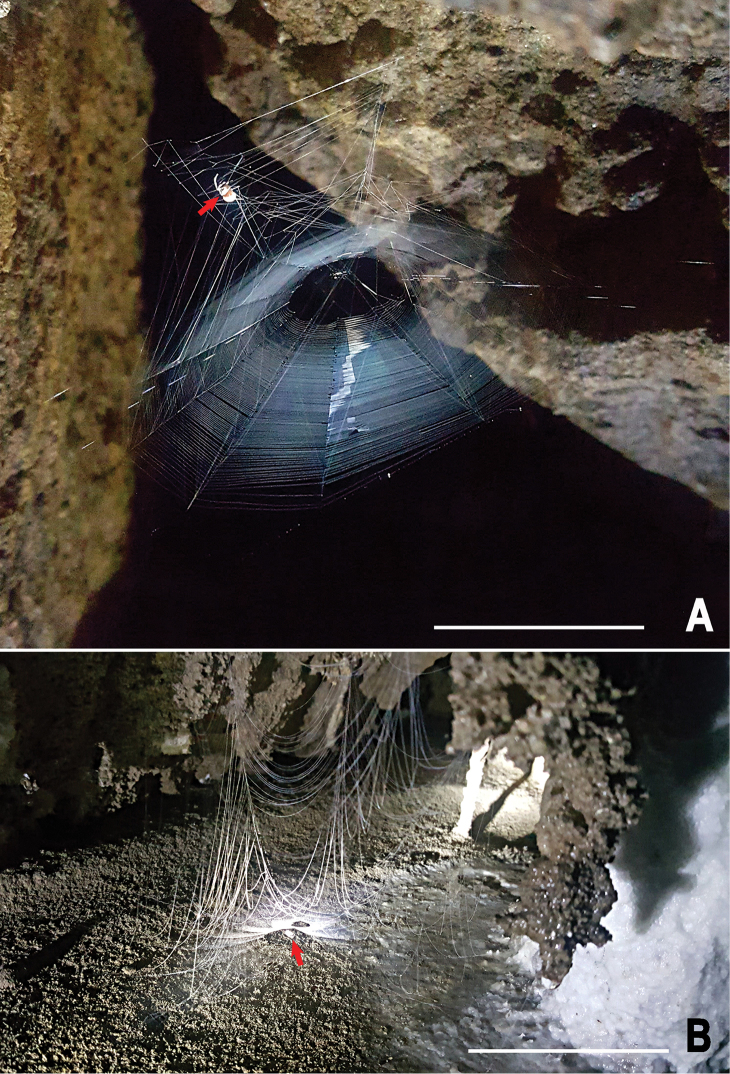
Webs of *Yamaneta* spiders in the Gaoligong Mountains **A***Yamaneta kehen* (Miller, Griswold & Yin, 2009) comb. nov. from Fugong Co., Lishadi, “a nameless cave”, female **B***Yamaneta paquini* (Miller, Griswold & Yin, 2009) comb. nov. from Lushui Co., Daxingdi, Walayaku [cave], female. Red arrows indicate location of spider. Scale bars: 20.0 mm.

##### Description.

**Male**. Somatic coloration and characters see Fig. 4A–C.

*Measurements*: Total length 2.19. Carapace 1.13 long, 1.12 wide. Clypeus 0.26 high. Sternum 0.57 long, 0.58 wide. Abdomen 1.09 long, 1.10 wide. Length of legs: I 6.98 (2.13, 0.66, 1.77, 1.27, 1.15); II 5.92 (1.83, 0.57, 1.46, 1.12, 0.94); III 3.93 (1.28, 0.39, 0.86, 0.74, 0.66); IV 4.25 (1.42, 0.40, 0.97, 0.83, 0.63).

*Male palp* (Fig. 5A–F): Femur long, curved mesially (Fig. 5E, F); patella elongate, with a distal-dorsal spine (Fig. 5E, F); tibia swollen, longer than patella, bears cluster of stiff dorsal setae and a dorsal trichobothrium proximally, with a long ventral setae distally (Fig. 5B: TiS). Cymbium broad, covers ventral part of bulb, dorsal part exposed (Fig. 5A–C). Paracymbium with long thick setae (Fig. 5C). Cymbial apophysis small hooked, sclerotized, on prolateral surface of cymbium (Fig. 5A–C). Cymbial conductor translucent, falcate, bearing dense cluster of long setae on prolateral face (Fig. 5C). Tegulum smooth, without process; spermatic duct long, twisted on base of embolus (Fig. 5D). Embolus long, wire-like, with proximal origin (Fig. 5B, D).

##### Female.

See Fig. 4D–F. Somatic characters as in male, but larger in size.

*Measurements*: Total length 2.48. Carapace 1.12 long, 1.10 wide. Clypeus 0.25 high. Sternum 0.64 long, 0.63 wide. Abdomen 1.43 long, 1.30 wide. Length of legs: I 6.46 (1.95, 0.63, 1.65, 1.21, 1.02); II 5.55 (1.66, 0.61, 1.38, 1.05, 0.85); III 3.82 (1.22, 0.42, 0.84, 0.73, 0.61); IV 4.09 (1.44, 0.40, 0.93, 0.75, 0.57).

*Vulva* (Fig. 6A–D): Scape relatively wide basally (Fig. 6A, C), with distinctly notched lateral margins (Fig. 6D) and strongly curved dorsal profile (Fig. 6B). Lateral margins of scape proximal to notches are converging (Fig. 6C, D).

##### Distribution.

Known from a single cave in Yunnan, China.

##### Natural history and web architecture.

This species lives in the dark zone of the cave. They build a web typical of *Maymena* (e.g., Eberhard 1986, Lopardo and Hormiga 2015: fig. 147D, E). Aerial lines extend upwards from web radii and hub and are attached to frame lines or the substrate above. The web is under tension and the hub is lifted. The catching spiral is dense and nearly horizontal. Above the catching spiral is an irregular network of horizontal and angled lines under tension (Fig. 10A). The spider usually hangs in the irregular area above the catching spiral.

#### Yamaneta paquini

Taxon classificationAnimaliaAraneaeMysmenidae

(Miller, Griswold & Yin, 2009) comb. nov.

F8BBB30A47075FA1A569B666D3BA84A9

Figs 7 , 8 , 9 , 10B

##### Material examined.

CHINA • 2♂♂ 3♀♀ 2 juveniles; Yunnan Province, Nujiang Lisu Autonomous Prefecture, Lushui County, Daxingdi Town, Walayaku [cave]; 26.13198N, 98.86149E; 940 m a.s.l.; 24 June 2016; Y.C. Li leg.; NHMSU • 2♂♂ 20♀♀ multiple juveniles; same data as for preceding; 18 Aug. 2018; Y.C. Li, Y. Li, Y.F. Shu & Y.C. Lin leg.; NHMSU • 1♂; same data as for preceding; GenBank: MK908794, MK908810, MK908802, MK895535, MK895543; GlgMY16 male • 1♀; same data as for preceding; GenBank: MK908795, MK908811, MK908803, MK895536, MK895544; GlgMY16 female • 1 juvenile; same data as for preceding; GenBank: MK908796, MK908812, MK908804, MK895537, MK895545; GlgMY16 juv.

##### Diagnosis.

See *Y. kehen*.

##### Description.

**Male**. Somatic characters see Fig. 7A–C, and Miller et al. 2009: 56.

*Measurements*: Total length 2.22. Carapace 1.10 long, 1.00 wide. Clypeus 0.25 high. Sternum 0.58 long, 0.60 wide. Abdomen 1.13 long, 0.99 wide. Length of legs: I 6.95 (2.10, 0.66, 1.79, 1.25, 1.15); II 5.88 (1.82, 0.57, 1.45, 1.12, 0.92); III 3.96 (1.31, 0.39, 0.86, 0.74, 0.66); IV 4.24 (1.42, 0.40, 0.96, 0.83, 0.63).

*Male palp* (Fig. 8A–F): Femur long, curved mesially (Fig. 8E, F); patella elongate, with a distal-dorsal spine (Fig. 8E, F); tibia swollen, longer than patella, bearing dense stiff dorsal setae and 2 dorsal trichobothrium proximally, 2 long ventral setae distally (Fig. 8A: TiS). Cymbium broad, covers ventral part of bulb, dorsal part exposed (Fig. 8A–C). Paracymbium with long thick setae (Fig. 8C). Cymbial apophysis small hooked, sclerotized, on prolateral surface of cymbium (Fig. 8A–C). Cymbial conductor translucent, falcate, bearing dense cluster of long setae on prolateral face (Fig. 8C). Tegulum smooth, without process; spermatic duct long, twisted on base of embolus (Fig. 8D). Embolus long, wire-like, with proximal origin (Fig. 8B, D).

##### Female.

Somatic characters see Fig. 7D–F, and Miller et al. 2009: 56.

*Measurements*: Total length 2.48. Carapace 1.16 long, 1.12 wide. Clypeus 0.25 high. Sternum 0.64 long, 0.63 wide. Abdomen 1.43 long, 1.30 wide. Length of legs: I 6.66 (1.96, 0.64, 1.64, 1.31, 1.11); II 5.81 (1.73, 0.62, 1.36, 1.13, 0.97); III 3.98 (1.27, 0.40, 0.85, 0.78, 0.68); IV 4.69 (1.50, 0.66, 1.03, 0.85, 0.65).

*Vulva* (Fig. 9A–D): Scape relatively narrow basally (Fig. 9A, C), with gently notched lateral margins (Fig. 9D) and moderately curved dorsal profile (Fig. 9B). Lateral margins of scape proximal to notches are nearly parallel (Fig. 9C, D).

##### Distribution.

Known from a single cave in Yunnan, China.

##### Natural history and web architecture.

This species lives in the dark zone of the cave. The web documented in Fig. 10B resembles that of *Y. kehen*, except that it is under less tension, causing lines in the superstructure to bow. The occupant of this web was observed below the catching spiral.

## Supplementary Material

XML Treatment for Yamaneta

XML Treatment for Yamaneta kehen

XML Treatment for Yamaneta paquini

## Supplementary materials

Supplementary material 1 Alignment of DNA sequence data used in phylogenetic analyses Data type: molecular data Explanation note: Contains plain alignments in Fasta (FengetalAlignment.fas) and Nexus formats (FengetalAlignment.nex), plus the Nexus file used for data partition and tree search in MrBayes (FengetalAlignmentMrB.nex). https://binary.pensoft.net/file/323857This dataset is made available under the Open Database License (http://opendatacommons.org/licenses/odbl/1.0/). The Open Database License (ODbL) is a license agreement intended to allow users to freely share, modify, and use this Dataset while maintaining this same freedom for others, provided that the original source and author(s) are credited.Chengcheng Feng, Jeremy A. Miller, Yucheng Lin, Yunfei Shu

Supplementary material 2 Uncorrected pairwise distances based on full alignment Data type: molecular data Explanation note: Contains uncorrected pairwise distances among all terminals as calculated using Mega X (Kumar et al. 2018). https://binary.pensoft.net/file/323858This dataset is made available under the Open Database License (http://opendatacommons.org/licenses/odbl/1.0/). The Open Database License (ODbL) is a license agreement intended to allow users to freely share, modify, and use this Dataset while maintaining this same freedom for others, provided that the original source and author(s) are credited.Chengcheng Feng, Jeremy A. Miller, Yucheng Lin, Yunfei Shu
